# Optical coherence tomography-derived coronary vessel wall abnormalities in adults long after Kawasaki disease

**DOI:** 10.1371/journal.pone.0342987

**Published:** 2026-02-25

**Authors:** Hiroyuki Ohashi, Yoshihide Mitani, Mitsuyasu Terashima, Toshiki Sawai, Hirofumi Sawada, Hidetoshi Hayakawa, Noriko Yodoya, Kaoru Dohi, Kakuya Kitagawa, Hajime Sakuma, Masahiro Hirayama

**Affiliations:** 1 Department of Pediatrics, Mie University Graduate School of Medicine, Mie, Japan; 2 Department of Cardiovascular Medicine, Toyohashi Heart Center, Aichi, Japan; 3 Department of Cardiology, Saiseikai Matsusaka General Hospital, Mie, Japan; 4 Department of Cardiology and Nephrology, Mie University Graduate School of Medicine, Mie, Japan; 5 Department of Advanced Diagnostic Imaging, Mie University Graduate School of Medicine, Mie, Japan; 6 Department of Radiology, Mie University Graduate School of Medicine, Mie, Japan; Universidade de Lisboa Instituto Superior Tecnico, PORTUGAL

## Abstract

**Background:**

Children with a history of Kawasaki disease (KD) and severe coronary involvement are at risk for acute coronary syndrome later in adulthood even in the absence of severe luminal lesions. We therefore investigated whether the coronary vessel walls in such adults are accompanied by potential substrates for acute coronary syndrome using optical coherence tomography (OCT), a high-resolution imaging modality.

**Methods:**

OCT was performed in patients who were followed up by serial coronary angiogram (CAG) and cardiac multi-detector computed tomography (MDCT) for ≥ 15 years after the diagnosis of acute KD with coronary artery aneurysms (≥ 6 mm in diameter).

**Results:**

Eleven patients (6 males, 55%) with median age 25.3 years (IQR: 22.7–30.3) and median interval 22.6 years (19.9–25.8) after acute KD were recruited. We investigated 51 coronary segments, comprising 43 coronary artery lesions (CALs) (19 regressed aneurysms, 37.2%; 16 persistent aneurysms, 31.4%; and 8 localized stenoses, 15.7%) and 8 normal segments (15.7%). OCT findings revealed fibrocalcific plaque in 20 segments (39.2%), fibroatheroma in 16 (31.4%), superficial signal-rich regions with attenuation in 14 (27.5%), microvessels in 18 (35.3%), luminal thrombi in 13 (25.5%), and ruptured plaque in 4 (7.8%). Qualitatively, all but one normal segment showed no OCT-derived abnormalities, whereas CALs, including regressed aneurysms, exhibited fibrocalcific plaques, fibroatheroma, and microvessels, along with luminal thrombi and ruptured plaques. Quantitatively, CAG-derived advanced lesions (persistent aneurysms and localized stenoses) and MDCT-derived calcified plaques were associated with OCT-detected vessel wall abnormalities.

**Conclusions:**

The present study showed that CALs in adults long after acute KD with severe coronary involvement are associated with OCT-derived vessel wall abnormalities, which are correlated with luminal lesions and MDCT-detected calcified plaques. Although these results do not demonstrate causality and may not be generalizable to milder cases, they warrant further studies to optimize screening and monitoring of adult KD-related coronary sequelae.

## Introduction

Kawasaki disease (KD) is an acute febrile illness characterized by coronary vasculitis, primarily affecting infants and young children. Up to 25% of untreated KD patients develop coronary aneurysms, posing significant risks for coronary events [1]. In Japan, over 298,000 patients have been diagnosed with KD, with 136,000 reaching adulthood [2], while in the U.S., over 24,000 young adults have a history of KD [3]. Adult KD patients with coronary sequelae often exhibit structural and functional coronary abnormalities [4–10], and studies have linked these long-term sequelae to acute myocardial infarction and sudden death [11–13].

Acute myocardial infarction in KD has previously been reported within 2 years post-illness, particularly in cases with giant aneurysms [14]. However, recent findings indicate young adults may experience their first event of acute coronary syndrome (ACS) remote after KD onset ascribed to segments even with regressed aneurysms or mild dilatation [15–18]. This suggests progressive coronary vessel wall abnormalities may underlie ACS risk long after the acute phase. However, the precise vessel wall substrate responsible for ACS in these cases remains unclear.

Optical coherence tomography (OCT) was shown to be useful for detecting thrombotic substrates and related abnormalities, such as ruptured plaque and fibroatheroma, in atherosclerotic patients [19–21]. In pediatric or adolescent KD patients, OCT studies have revealed intimal thickening, elastic lamina fragmentation, and calcified lesions [22–26]. However, whether any substrates potentially responsible for ACS are present in adults ≥15 years after KD onset in cases with severe coronary artery lesions (CALs), is unknown. In the present study, we investigated whether OCT-derived vessel wall abnormalities are found in adult KD patients with severe coronary involvement.

## Methods

### Study population

We analyzed consecutive KD patients who underwent OCT at Mie University Hospital from 09/11/2012–30/11/2021. Inclusion criteria were: (1) clinical diagnosis of KD, (2) an interval ≥ 15 years from disease onset, (3) CALs ≥ 6 mm in diameter in any segment during or shortly after the acute illness as evaluated by cardiac ultrasonography or coronary angiography (CAG), (4) any CALs identified by CAG prior to OCT, and (5) regular follow-up from KD onset in accordance with Japanese Circulation Society/Japanese Society for Cardiovascular Surgery (JCS/JSCS) guidelines [2]. We excluded patients with coronary angiographic findings: (i) ≥ 75% stenotic lesion, (ii) severe vessel tortuosity and poor runoff of the contrast medium in the vessels, and (iii) clinical or hemodynamic instability at CAG. CALs were classified according to the JCS/JSCS guidelines [2], with CALs determined by cardiac ultrasonography or CAG in acute and convalescent phases and CAG before OCT. The study was approved by the Mie University Graduate School of Medicine ethics committee (approval no. 2456), and written informed consent was obtained from all patients or their parents.

### Acquisition and analysis of OCT

During the procedure, heparin was given as a bolus of 3000 U with additional boluses to 1000 U/h. After the completion of the diagnostic coronary angiography under the local anesthesia, a 3.2 F second-generation frequency domain C7-XR system (*frequency domain*-OCT Imaging System, St. Jude Medical, St. Paul, Minnesota, USA) was placed through a 6F guiding catheter at a distal portion in the right and the left main trunk, left anterior descending, or left circumflex coronary arteries when possible. Imaging was performed by automated pullback triggered at a speed of 20 mm/s. OCT routinely captures 54 mm of the vessel on each pullback, and this is divided into 256 frames. This was performed simultaneous injection of 10–15 ml of contrast by power injector through the guiding catheter. The images were saved and sent to an independent OCT server, which was blinded to patient characteristics. Qualitative OCT image analysis was performed by two independent investigators who were blinded to the clinical presentation (HO, MT). For any disagreement in data evaluation between the readers, consensus agreement was achieved.

All cross-sectional images underwent initial quality review, with frames excluded if a side branch occupied > 45 degrees of the cross-section or if the image exhibited poor quality due to residual blood, artifact, or reverberation. The plaque characteristics were defined in accordance with previously validated criteria [19–21]. Fibrous plaques are defined by homogeneous high back-scattering areas. Fibroatheroma plaques are defined by signal-poor OCT regions with poorly delineated borders. Fibrocalciﬁc plaques are defined by fibrous tissue intermixed with calcium, appearing as signal-poor or heterogeneous regions with well-defined borders. Superficial Signal-Rich Regions with Attenuation (SSR) are defined by signal-rich regions surpassing background speckle noise intensity. Microvessels are defined by sharply delineated signal-poor voids, often traceable across multiple contiguous frames within the plaque. Ruptured plaques are defined by intimal tearing, disruption, or dissection of the fibrous cap. Thrombi are defined by an abnormal mass protruding into the coronary lumen with signal backscattering and varying degrees of attenuation, adhering to the arterial wall [21].

Based on previous studies with histological validations, the intima was defined as a signal-rich layer nearest to the lumen, and the media was defined as a signal-poor middle layer. The internal elastic lamina was defined as a signal-rich band between the intima and media, and the external elastic lamina was defined as a signal-rich band between the media and adventitia [21]. In the present study, maximum intimal diameter was measured. At persistent aneurysm (PAN) segments, if the lumens of which were too large to visualize by OCT, the measurements were made in sections adjacent to the aneurysm. Measurements of the intima were conducted by the cardiologist (HO) performing CAG with OCT. In accordance with the previous study [25], we defined thickness > 400 μm as abnormal.

Lesion morphology was assessed per segment by cardiologists (HO, YM, MT). Major vessel wall abnormalities included fibrocalcific plaque, fibroatheroma, SSR, microvessels, luminal thrombi, and ruptured plaque. Minor abnormalities were defined as intimal thickness > 400 μm [25] or medial irregularity.

### Acquisition and analysis of data from multi-detector computed tomography

Image acquisition was performed using a dual-source CT scanner (SOMATOM Definition Flash or SOMATON Force, Siemens Healthcare, Erlangen, Germany). For calcium scoring, a prospective electrocardiogram (ECG)-gated scan was performed at 75% of the R-R interval. Subsequently, prospectively ECG-triggered high-pitch spiral acquisition was performed with injection of 0.84 ml/kg of iopamidol over 12 seconds. The scan parameters were 70 kV tube voltage and 0.28-second gantry rotation time with contrast medium, and scans were performed during a single breath-hold. After the acquisition, the data were reconstructed and transferred to a remote workstation for post-processing. The multi-detector computed tomography (MDCT) angiograms were evaluated by two experienced observers who were unaware of the clinical characteristics. Disagreements were immediately resolved in consensus. The coronary artery was assessed in each coronary segment. To accurately match the OCT images, side branches and coronary ostia were used as landmarks, and contrast-enhanced images were viewed for verification of the segmentation. The presence of coronary plaque was visually evaluated based on the contrast-enhanced coronary angiograms using axial images and curved multiplanar reconstructions. Coronary plaque is defined as structures > 1 mm^2^ within and/or adjacent to the coronary artery lumen, which is clearly distinguished from the vessel lumen and the surrounding tissue. Coronary plaque is visually classified as follows: 1) non-calcified plaque having lower density compared with the contrast-enhanced vessel lumen present without any calcification discernible; 2) calcified plaque with areas above one hundred thirty Hounsfield unit. Calcified plaques were identified as contiguous structures > 130 Hounsfield units (HU) in at least four adjacent sections, with density values ranging from −50–750 HU [27,28]. Two independent radiologists (KK, H Sakuma), who were blinded to clinical history, evaluated reconstructed images.

### Imaging coregistration and segment analysis of OCT and MDCT

All 15 coronary artery segments according to the classification of the American Heart Association [29] with a diameter of 1.5 mm or more on conventional coronary angiography constituted the basis of analysis [30]. OCT and MDCT images were matched by using reproducible vessel landmarks as anatomical axial references. Visible landmarks through the target vessel length were annotated for both OCT and MDCT, and all vessel portions were carefully scrutinized to match the exact counterpart.

### Acquisition and analysis of data from perfusion magnetic resonance imaging

First-pass contrast-enhanced myocardial perfusion images were acquired during adenosine triphosphate (ATP) stress and rest using a 1.5-T clinical MRI scanner (Achieva 1.5 T, Philips Medical Systems, Best, the Netherlands) with a 5-channel cardiac receiver coil. Image analysis was conducted using a commercially available post-processing workstation (CMR42, Circle Cardiovascular Imaging Inc., Calgary, Canada). Assessment of LV regional wall motion abnormalities in the 16-segment model. Stress-rest first-pass myocardial perfusion MR images were further analyzed utilizing an image analysis workstation (Virtual Place, Aze, Tokyo, Japan). The left ventricular (LV) myocardium was divided into 16 segments, comprising 6 basal segments, 6 mid-ventricular segments, and 4 apical segments based on the AHA 17-segment model, with exclusion of the apical segment.

Myocardial perfusion MR images were acquired with steady-state perfusion MR sequence with non-slice-selective saturation recovery preparation (four short-axis imaging slices, two images per heartbeat, 3.0 ms repetition time [TR], 1.2 ms echo time [TE], 45 degrees flip angle and, 150 ms between the saturation preparation pulse and the center of k-space acquisition, 36x32 cm field of view, 128x128 acquisition matrices and 8 mm section thickness). For both stress and rest perfusion MR imaging, gadolinium contrast medium (gadopentetate dimeglumine, Magnevist, Schering, Berlin, Germany) was injected into the right antecubital vein at a dose of 0.05 mmol kg^-1^ with a power injector at a flow rate of 4 ml s^-1^, followed by a 20-ml saline flush. Dynamic MR images were acquired for 1 minute. Pharmacological stress was performed by injecting ATP (160 mg kg^-1^ min^-1^) in the left antecubital vein for 4 minutes. At 3 minutes after starting ATP administration, the acquisition of stress myocardial perfusion MR images was initiated and ATP was continuously injected during the acquisition of the stress perfusion MR images. Rest myocardial perfusion MR images were acquired at least 10 minutes after finishing the stress myocardial perfusion MR image. Immediately after rest myocardial perfusion MR images, an additional gadolinium dose was injected to reach a cumulative dose of 0.15 mmol kg^-1^. Then, 10–15 minutes later, late gadolinium enhancement was obtained on short- and long-axis imaging planes of the left ventricle by using an inversion recovery 3D turbo field-echo (TFE) sequence (3.8 ms TR, 1.2 ms TE, flip angle = 15°, field-of-view = 40x36x5 cm, acquisition matrix size = 224x156x5, reconstructed matrix size = 256x256x10, SENSE factor = 2, TFE-factor = 24). Inversion time was adjusted in each patient to null signal from the normal myocardium by using a look-locker sequence. Inducible myocardial perfusion defects were visually identified on three short-axis images (at the basal, mid-ventricular, and apical levels) as areas of subendocardial/transmural hypoperfusion appearing during the first pass of contrast and persisting for > 4 RR intervals. These defects were required to be more than 1 pixel wide and to conform to the distribution territory of one or more coronary arteries [31,32].

The presence or absence of inducible myocardial perfusion defects was classified as positive or negative ischemia. Infarcted myocardium was defined when the signal intensity exceeded 5 standard deviations above remote normal myocardium in late gadolinium enhancement (LGE) images [33]. Viability was defined as a transmural extent of hyperenhancement < 50% [34]. Myocardial infarction was categorized as negative, subendocardial myocardial infarction (SEMI), defined as < 50% LGE of myocardial thickness based on visual assessment, or transmural myocardial infarction (TMI), defined as > 50% LGE of myocardial thickness based on visual assessment. Two experienced cardiac radiologists, blinded to patient history and follow-up, reviewed the images by consensus.

### Statistical analysis

Data were managed and analyzed using SPSS PASW Statistics (v27.0), R, and EZR (Jichi Medical University). Quantitative OCT findings were analyzed using estimation-focused statistical methods due to the clustered observations and relatively small sample size. Data management and statistical analyses were performed with SPSS Inc. PASW Statistics for Windows, Version 27.0. Chicago: SPSS Inc., program R (http://cran.r-project.org) or EZR (Saitama Medical Center, Jichi Medical University), which is a graphical user interface for R (The R Foundation for Statistical Computing) [35]. Continuous data are reported throughout the text as median and interquartile range when appropriate. Categorical data are expressed as the frequency of occurrence. The diagnostic accuracy (sensitivity, specificity, positive, and negative predictive values including 95% confidence intervals) of CAG-derived advanced luminal findings and CT-derived calcified plaque for the detection of OCT-derived atherosclerotic intima (fibrocalcific plaque, fibroatheroma and microvessels) was calculated on segmental basis. OCT served as the reference standard for detecting atherosclerotic intima. Segments were classified as true positive if MDCT correctly identified calcified plaque or if CAG accurately detected PAN or localized stenoses (LS) lumen abnormalities. These confidence intervals (CIs) are adjusted for clustering in the data by dividing the sample size by a design effect (which was computed as a function of the average number of ROI in each artery and the intracluster correlation coefficient) to obtain an effective sample size on which intervals are based.

## Results

### Patient and lesion characteristics

**Fig 1:**
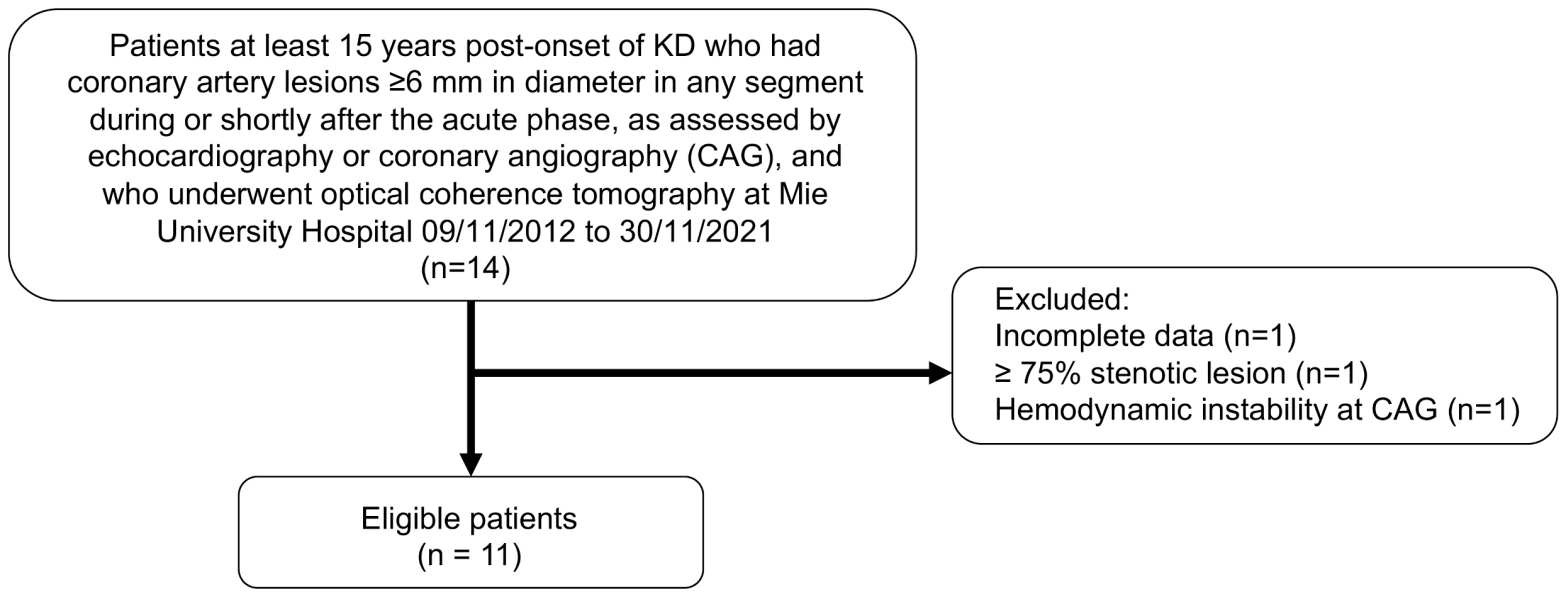
A flow diagram of patient selection. KD indicates Kawasaki disease.

We reviewed 44 consecutive patients with KD who underwent CAG at Mie University Hospital from 09/11/2012–30/11/2021. Of these, 14 patients who met the inclusion criteria underwent OCT evaluation. After excluding three patients based on the exclusion criteria, 11 Japanese patients (6 males and 5 females) met the eligibility criteria and were included for analysis (Fig 1). The median age at the time of the OCT study was 25.3 years (IQR: 22.7–30.3 years; range: 16.3–32.9 years), with a median interval of 22.6 years (IQR: 19.9–25.8 years; range: 15.5–32.1 years) from the acute phase of KD. Table 1 summarizes the demographic details, coronary angiographic findings, and the number of segments with CT-derived atherosclerotic plaque for each patient. None of the patients had conventional risk factors for ischemic heart disease, such as dyslipidemia, hypertension, or diabetes mellitus, except for one patient classified as obese (body mass index > 30 kg/m²), one with a smoking history, and one with a family history of ischemic heart disease. All patients presented with PAN, LS, or occlusions in one or more coronary segments in the CAG conducted immediately before the OCT study. One patient had a history of silent myocardial infarction attributed to thrombotic occlusion of a right coronary artery aneurysm (7.8 mm in diameter) several years after acute KD, which was confirmed by elective MR imaging. Another patient experienced ACS at the age of 23, necessitating percutaneous coronary intervention. None of the patients had undergone coronary artery bypass graft surgery. All patients were on antiplatelet therapy, but none were receiving anticoagulants at the time of the OCT study.

**Table 1:** Patients characteristics.

						CAG Findings		CT Findings	MRI Findings	
Patient	Sex	Age, y	Age at KD, y	CV Risk Factors	Cardiac Event	LCA	RCA	Number of segments with atherosclerotic plaque	LGE	Stress Perfusion
1	M	21.2	2.2			RAN, LS	RAN, LS	Calcified Plaque 3, NC Plaque 0	Negative	Negative
2	F	26.2	3.6	Obesity		PAN	NS	Calcified Plaque 1, NC Plaque 0	Negative	
3	M	16.3	0.8		SEMI	RAN, PAN, LS	PAN	Calcified Plaque 3, NC Plaque 0	SEMI	Negative
4	M	24.0	5.3	FH		RAN, PAN, LS	PAN	Calcified Plaque 3, NC Plaque 0	Negative	
5	M	25.3	2.2			RAN	PAN	Calcified Plaque 0, NC Plaque 0	Negative	
6	M	31.6	9.0		SMI	PAN, LS	OC	Calcified Plaque 1, NC Plaque 0	TMI	Negative
7	F	30.7	1.4			PAN, LS	PAN, LS	Calcified Plaque 2, NC Plaque 3	Negative	Negative
8	M	23.7	0.7	Smoking	ACS	RAN	RAN, PAN	Calcified Plaque 2, NC Plaque 1	TMI	Negative
9	F	29.9	1.4			PAN	RAN	Calcified Plaque 1, NC Plaque 1	Negative	Negative
10	F	21.7	4.9			RAN, PAN	RAN	Calcified Plaque 1, NC Plaque 1	(ND)	(ND)
11	F	32.9	0.8			NS	PAN	Calcified Plaque 1, NC Plaque 0	Negative	

CV indicates cardiovascular; CAG, coronary angiography; SMI, LCA, left coronary artery; RCA, right coronary artery; CT, computed tomography; MRI, magnetic resonance imaging; LGE, late gadolinium enhancement; FH, family history of heart disease; SEMI, subendocardial myocardial infarction, SMI, silent myocardial infarction; ACS, acute coronary syndrome; NS, normal coronary segment; RAN, regressed aneurysm; PAN, persistent aneurysm; LS, localized stenosis; OC, occlusion; NC, non-calcified; TMI, transmural myocardial infarction; and ND, not determined.

Eight normal segments (three of segment 3, two of segment 7, two of segment 11 and one of segment 13) from the disease onset, that were determined as normal by CAG < 6months after the acute illness, were included for the analyses. Stenotic lesions (< 75%) in 5 patients were included, who exhibited no ischemic symptoms or abnormalities in clinical tests, including myocardial perfusion MR imaging and exercise electrocardiogram.

### OCT findings

**Fig 2:**
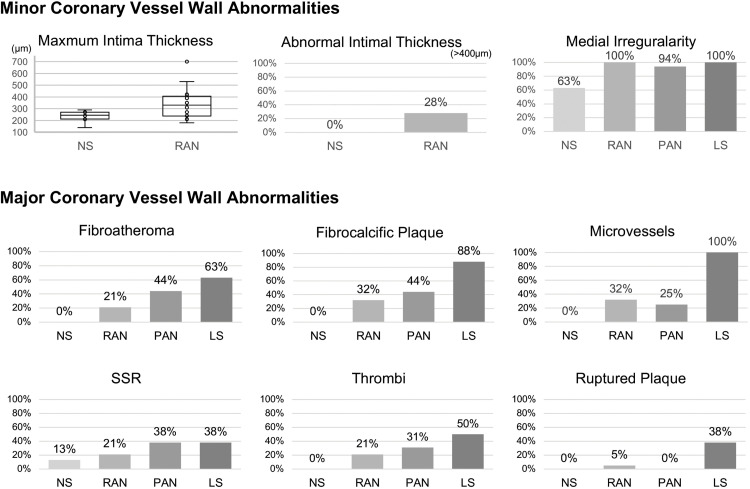
OCT findings in coronary artery lesions. NS indicates normal coronary segment; RAN, regressed aneurysm; PAN, persistent aneurysm; LS, localized stenosis; and SSR, superficial signal-rich regions with attenuation.

A total of 54 coronary segments in 11 patients were analyzed using OCT. Two segments were excluded for the OCT analysis because of the artifacts. One segment that had undergone percutaneous coronary intervention with stenting was also excluded. The final analysis was performed in 51 segments (8 normal segments and 43 segments with CALs). The right coronary artery was evaluated in 10 out of 11 patients, the left main trunk and left anterior descending artery in all 11 patients, and the circumflex artery in 10 out of 11 patients. Each patient had a median of five segments analyzed (IQR 3–6). Minor and major coronary vessel wall abnormalities were described by the luminal lesion below (Fig 2). Representative imaging findings are presented in , and a summary of these cases is provided in S1 Table.

**Fig 3:**
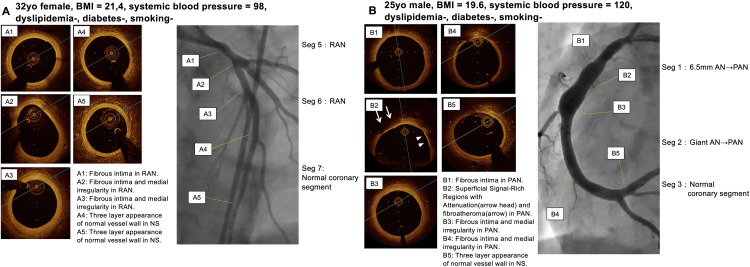
Representative imaging findings. Representative OCT images in no coronary lesions form the onset of disease (A4, A5, B5), regressed aneurysm segments (A1-3) and persistent aneurysm segments (B1-4) from 2 patients long after KD (A, patient 11; B, patient 5).

**Fig 4:**
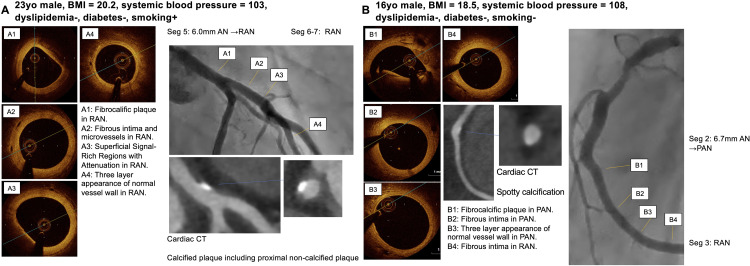
Representative imaging findings. Representative OCT and MDCT images in regressed aneurysm segments (A1-4), persistent aneurysm segments (B1-4) and from 2 patients long after KD (A, patient 8; B, patient 3).

**Fig 5:**
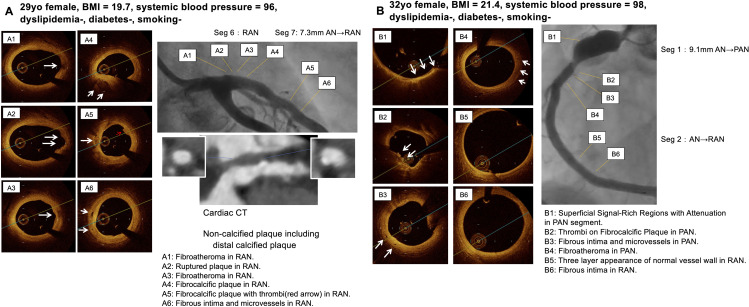
Representative imaging findings. Representative OCT and MDCT images in regressed aneurysm segments (A1-5, B5, B6) and persistent aneurysm segments (B1-4) from 2 patients long after KD (A, patient 9; B, patient 11).

**Fig 6:**
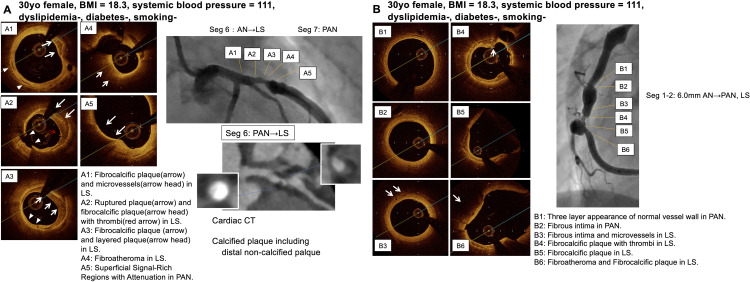
Representative imaging findings. Representative OCT and MDCT images in persistent aneurysm. segments (A5, B6) and localized stenosis segments (A1-4, B1-5) from a female patient without risk factor (patient 7).

#### Normal coronary arterial segments from the onset.

All eight normal segments had a maximum intimal thickness of less than 400 μm, with a median thickness of 245 μm (IQR: 218–270 μm). We observed medial irregularity in five of the eight normal segments (63%) (Fig 2). Among these eight normal segments, only one (13%) exhibited a major coronary vessel wall abnormality, classified as SSR. In all the normal segments examined, the arterial wall structure consistently revealed three distinct layers: the internal elastic lamina (a bright, highly reflective abluminal line), the media (a dark, low-reflective line), and the external elastic lamina (a highly reflective abluminal zone).

#### Regressed aneurysms.

We investigated OCT-derived intimal lesions in 19 regressed aneurysms. The maximum intima thickness, which was determined in 18 segments, was 330 μm (median; IQR, 248–398). Of note, 5 out of 18 regressed aneurysm lesions (28%) exhibited intimal thickness over 400μm. Medial irregularity was observed in all segments (Fig 2). Twelve segments in 19 regressed aneurysms (63%) exhibited any of major coronary vessel abnormalities. Fibrous plaques were the predominant finding in regressed aneurysm. The three-layered appearance was also observed in any parts of all the regressed aneurysms. The major coronary wall abnormalities included fibrocalcific plaque, fibroatheroma, microvessels, protruding mass regarded as thrombi and ruptured plaque (Fig 2). Patient 9 with the ruptured plaque did not exhibit late gadolinium enhancement in MR imaging.

#### Persistent aneurysms.

Of the 16 persistent aneurysms analyzed, all except one exhibited severely thickened intima and medial irregularity (Fig 2). Fourteen out of 16 persistent aneurysms (88%) had any of major coronary vessel abnormalities, including thrombi on fibrocalcific plaque, fibroatheroma, SSR, and microvessels.

#### Localized stenotic lesions.

All eight stenotic lesions exhibited severely thickened intima, medial irregularity, and any of major coronary vessel wall abnormalities. These abnormalities were frequently observed in stenotic lesions (Fig 2). Coronary vessel wall abnormalities included fibrocalcific plaques, microvessels, thrombi on fibrocalcific plaque, ruptured plaques, fibroatheroma, and SSR.

### Quantitative analysis of OCT-derived intimal lesions in coronary artery lesions

**Fig 7:**
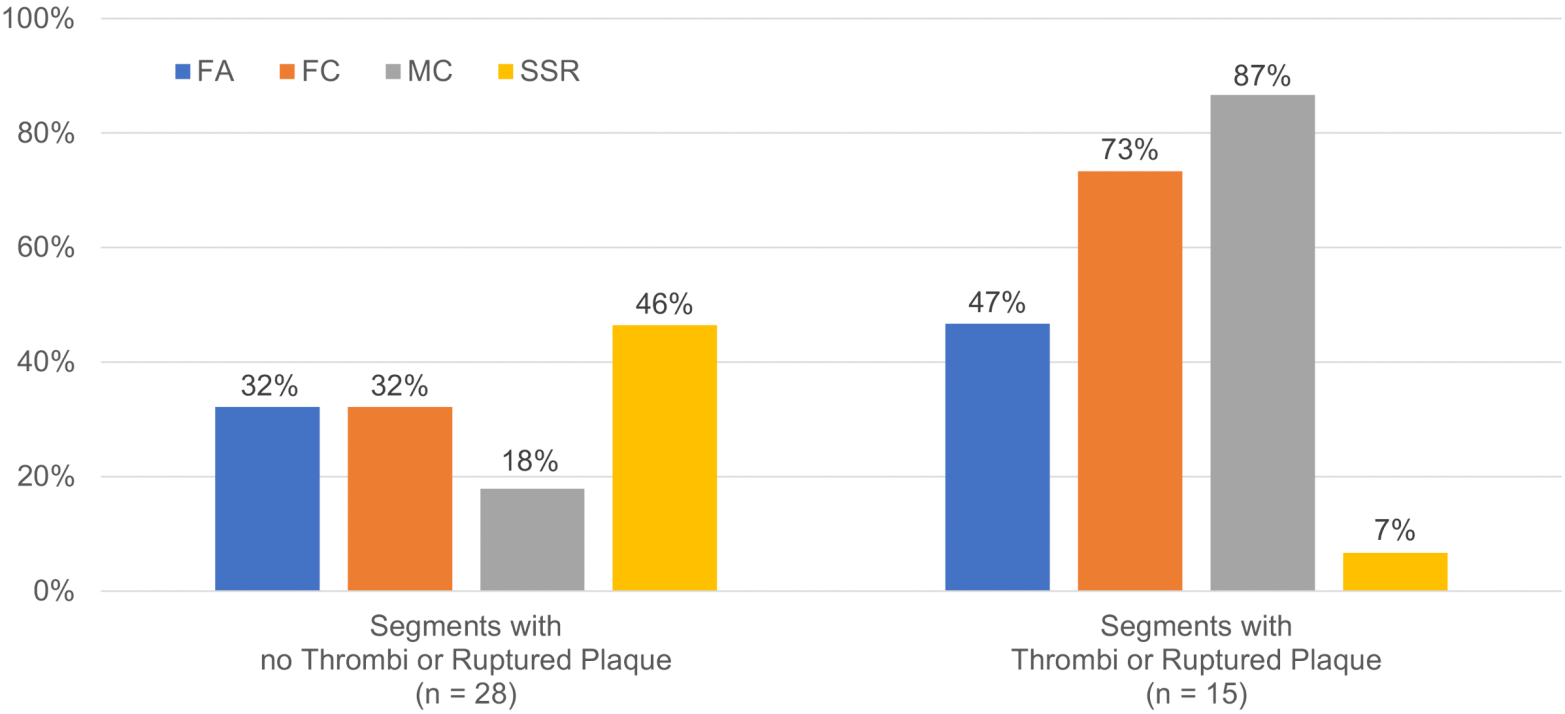
Plaque composition in various coronary lesions with no thrombi or ruptured plaque and in lesions with thrombi or rupture plaque. FA indicates fibroatheroma; FC, fibrocalcific plaque; MC, microvessels; and SSR, superficial signal-rich regions with attenuation.

A variety of OCT-derived intimal lesions were observed in CALs, including those in regressed aneurysms. CALs were more frequently associated with fibrocalcific plaques, fibroatheroma-like plaques, microvessels, and thrombi compared to normal segments. Microvessels were detected in all localized stenotic lesions. Luminal thrombi or ruptured plaques were more likely to be observed on fibrocalcific lesions and lesions with microvessels than on fibroatheroma (Fig 7). Specific cases were shown in S1 File.

### Diagnostic accuracy of CAG-derived advanced luminal findings and CT-derived calcified plaque for OCT findings

The diagnostic accuracy of CAG-derived PAN or LS lumen abnormalities and CT-derived calcified plaque (with 95% CIs) for detecting OCT-derived atherosclerotic intima (fibrocalcific plaque, fibroatheroma, and microvessels) was compared on a segmental basis and summarized in Table 2. MDCT-derived calcified plaque had higher negative and positive predictive values as well as accuracy for predicting fibrocalcific plaque and microvessels than luminal diagnostics. The correlation between MDCT-derived calcified plaque and CAG-derived luminal lesions is shown in S3 Fig.

**Table 2:** Comparison of diagnostic accuracy of CAG-derived advanced luminal findings and CT-derived calcified plaque for detection of OCT findings.

Variable	Lumen Diagnosis (PAN or LS)	CT-derived calcified plaque
For Detecting Fibroatheroma		
Sensitivity	12/16 (75 {48, 93})	10/16 (63 {35, 85})
Specificity	23/35 (66 {48, 81})	28/35 (80 {63, 92})
Negative predictive value	23/27 (85 {66, 96})	28/34 (82 {66, 93})
Positive predictive value	12/24 (50 {29, 71})	10/17 (59 {33, 82})
Accuracy	35/51 (69 {54, 81})	38/51 (75 {60, 86})
For Detecting Fibrocalcific Plaque		
Sensitivity	14/20 (70 {46, 88})	15/20 (75 {51, 91})
Specificity	21/31 (68 {49, 83})	29/31 (94 {79, 99})
Negative predictive value	21/27 (78 {58, 91})	29/34 (85 {69, 95})
Positive predictive value	14/24 (58 {37, 78})	15/17 (88 {64, 99})
Accuracy	35/51 (69 {54, 81})	44/51 (86 {74, 94})
For Detecting Microvessels		
Sensitivity	12/18 (67 {41, 87})	13/18 (72 {47, 90})
Specificity	21/33 (64 {45, 80})	29/33 (88 {72, 97})
Negative predictive value	21/27 (78 {58, 91})	29/34 (85 {69, 95})
Positive predictive value	12/24 (50 {29, 71})	13/17 (77 {50, 93})
Accuracy	33/51 (65 {50, 78})	42/51 (82 {69, 92})

## Discussion

The major findings in the present study are as follows: firstly, the present OCT study identified potential substrates for ACS, including fibrocalcific plaques, fibroatheroma-like plaques, and microvessels with or without thrombi or ruptured plaque in the coronary vessel walls of young adult patients long after Kawasaki disease (KD); secondly, normal segments exhibited few major coronary vessel wall abnormalities and only minor coronary vessel wall abnormalities such as medial irregularity; thirdly, advanced luminal lesions identified through CAG and calcified plaque detected by MDCT had a certain predictive value for OCT-derived vessel wall abnormalities. Overall, these findings suggest that severe CALs occurring shortly after acute KD are associated with OCT-detected major coronary vessel wall abnormalities in adulthood. The presence of these abnormalities also correlates with luminal lesions and MDCT-detected calcified plaque.

In the present study, we focused on adult KD patients (median age: 25.3 years) with a median interval of 22.6 years after acute KD, in contrast to previous studies conducted primarily in children [22–25]. In addition, the present KD patients were diagnosed with KD during acute illness and were shown to be complicated by CALs ≥ 6 mm in diameter in any segment immediately following the acute illness. They were regularly followed up, and cardiovascular complications were evaluated by CAG immediately prior to the OCT study, together with MDCT and cardiac MR imaging. The severity of CALs in the present population is distinct from those in a previous OCT study focusing on the segments that were normal from the onset or regressed aneurysms [22–26]. The presence of CALs ≥ 6 mm in diameter in the convalescence of KD was in line with the prospective culprit lesions in the adult KD ACS survey [36].

This is the first OCT study that uncovered the vessel wall abnormalities in adult KD patients with coronary sequelae but without myocardial ischemia, who may be at risk for ACS in adulthood. Our findings showed that in CALs including regressed aneurysms, OCT-derived abnormalities such as fibrocalcific plaques and microvessels were observed, while normal segments from the onset of KD exhibited no major abnormalities. These findings give an insight into the screening and monitoring of adult KD patients at the risks for ACS. The present OCT findings were consistent with the previous IVUS-based reports [7,37]. However, such OCT-derived abnormal findings, especially fibrocalcific plaques and microvessels, were accompanied by luminal thrombosis. In the case shown in S1 Fig, these findings appeared to be associated with MRI-derived silent myocardial infarction. Since most of the culprit lesions in the long-term of KD patients in the adult KD ACS survey were accompanied by any persistent aneurysms and/or localized stenoses [1,14,35,38,39], it is possible that such OCT findings may have a certain prognostic value for ACS in KD adult patients. Furthermore, CAG-derived severe luminal lesions and MDCT-derived calcified plaque had a certain predictive value for the presence of fibroatheroma, fibrocalcific plaque and microvessels. Therefore, these findings suggest that severe luminal lesions and MDCT-derived calcified plaque may give us an insight into the potential substrates for ACS in KD patients in adulthood.

Superficial signal-rich regions with attenuation were occasionally observed in a variety of luminal lesions in the present study. Although superficial signal-rich regions with attenuation were reported to represent macrophage infiltration in atherosclerotic patients in non-KD adults [21,40], its clinical relevance is unknown in KD. In fact, the presence of macrophage infiltration is still controversial in previous pathological studies in KD and such OCT findings were not previously validated pathologically [41–43]. Abnormal intimal thickening and medial irregularity were observed in normal segments and regressed aneurysms, that were consistent with the previous studies [23,25,26]. Since such OCT-derived minor coronary vessel wall abnormalities were not associated with luminal thrombi in the present study, their clinical relevance is unknown in the present study. OCT-derived fibroatheroma was less strongly associated with mural thrombi, compared to fibrocalcific plaque or microvessels. The contribution of atheroma to the coronary events were rarely reported in previous pathological reports in KD patients [41–43]. It is possible that a relatively small plaque volume of the fibroatheroma in the severely thick intima with multiple components, as shown in S2 Fig and previous IVUS-based reports [7], may not be sufficient enough to cause even MRI-derived myocardial infarction. Thus, the present findings of luminal thrombi found on fibrocalcific plaque or the plaques with microvessels may be consistent with the hypothesis that any erosion-like mechanisms may work for the thrombus formation in KD adults [15–18,44,45].

### Study limitations

Several limitations should be considered in interpreting the results. First, the quantitative findings in the present study were not analyzed using hypothesis testing because of the small sample size and the clustering of observations within patients. However, this is the first study to provide important OCT findings in a well-characterized adult KD population with severe coronary lesions. Second, no data were available to correlate OCT findings with pathological findings in the corresponding vessels of patients with KD, although the interpretation of OCT findings has been well validated in atherosclerotic or healthy human coronary arteries in vivo and ex vivo [21,46–48]. Third, the sample may be biased, as all patients investigated had had aneurysms ≥ 6 mm in diameter in the convalescence and had persistent aneurysms, localized stenoses in any segments at the time of investigation. Thus, OCT-derived vessel wall abnormalities may be milder in the adulthood with less severe lesions. Fourth, our findings represent descriptive associations and do not demonstrate causality with respect to ACS. Accordingly, the association between these vessel wall abnormalities and the occurrence of past or future ACS events remains undetermined. Fifth, computational fluid dynamics-based hemodynamic analysis was not performed. Future assessment of wall shear stress by such methods would strengthen the mechanistic interpretation of the OCT findings.

### Clinical implications

First, the present limited but initial OCT study in adults with KD-related severe coronary involvement uncovered OCT-derived vessel wall abnormalities suggesting potential substrates for ACS. Second, the normal segments from the disease onset were rarely accompanied by any such major vessel wall abnormalities, suggesting that such normal segments might not be at risk for ACS in this age group. Third, CAG-derived luminal lesions and MDCT-derived calcified plaque had a certain predictive value for OCT-derived abnormalities. Therefore, any modalities for evaluating such lesions, including MDCT, could be useful for the screening and monitoring of the risks for ACS in the adult KD population. Finally, future prospective cohort studies are warranted to validate the clinical impact of these monitoring strategies on long-term outcomes in this population.

## Conclusions

The present study showed that CALs including regressed aneurysms in adults long after acute KD with severe coronary involvement were accompanied by OCT-derived vessel wall abnormalities. These abnormalities were correlated with luminal lesions and MDCT-derived calcified plaques. These findings warrant further studies for optimizing the screening and monitoring of KD-related coronary sequelae in adulthood with the use of vessel wall imaging as well as assessments for induced ischemia.

### Clinical perspective

Acute myocardial infarction in Kawasaki disease (KD) patients was previously reported in children complicated by giant aneurysms early after the acute illness. Recent reports indicate that young adults, long after KD, experienced their first episode of acute coronary syndrome, with culprit lesions not always associated with giant aneurysms or severe stenoses. These findings suggest that any coronary vessel wall abnormalities which developed over time may contribute to acute coronary syndrome in young adult KD patients. Optical coherence tomography (OCT), a high-resolution imaging modality, can reveal substrates for thrombotic events and related abnormalities, akin to findings in atherosclerotic adults. We therefore investigated whether KD-related coronary artery lesions (CALs) are associated with OCT-derived potential substrates for acute coronary syndrome in adulthood. We found that CALs, including regressed aneurysms in adults long after acute KD, were accompanied by OCT-derived vessel wall abnormalities, which were related to luminal lesions and multi-detector computed tomography-derived calcified plaques. These findings highlight the need for further research to optimize screening and monitoring strategies for KD-related coronary sequelae in adulthood, emphasizing vessel wall imaging alongside evaluations of induced ischemia.

## Supporting information

S1 Table Supplemental table 1. Summary of representative cases. (DOCX)

S1 File Supplement I: Specific cases of luminal thrombi or ruptured plaques. Patient 3, who had thrombi on fibrocalcific plaque with microvessels and no atheroma in the left coronary artery, was accompanied by subendocardial infarction in cardiac MR imaging (S1 Fig). Patient 7, with a small ruptured plaque on fibroatheroma, had neither cardiac events nor MR-derived silent myocardial infarction in the corresponding segment (S2 Fig). In the MDCT study, the presence of MDCT-derived calcified plaque was in parallel with the severity of CAG-derived luminal lesions (S3 Fig).
(DOCX)

S1 Fig Supplemental figure 1. Patient 3, who had fibrocalcific plaque, microvessels, and thrombus in the left coronary artery and no fibroatheroma, was diagnosed with subendocardial infarction on cardiac MR imaging. PAN indicates persistent aneurysm; LS, localized stenosis.
(JPG)

S2 Fig Supplemental figure 2. Patient 7 had no cardiac event revealed by cardiac MR imaging, despite the presence of atherosclerotic-like lesions including fibroatheroma and ruptured plaque. PAN indicates persistent aneurysm; LS, localized stenosis.
(JPG)

S3 Fig Supplemental figure 3. CT-derived calcified plaque in coronary artery lesions. NS indicates normal coronary segment; RAN, regressed aneurysm; PAN, persistent aneurysm; LS and localized stenosis.
(TIF)
