# Supplementary material for: Optical coherence tomography-derived coronary vessel wall abnormalities in adults long after Kawasaki disease
Source: PLoS One. 2026 Feb 25;21(2):e0342987. doi: 10.1371/journal.pone.0342987 (PMC12935235; doi:10.1371/journal.pone.0342987)
Supplement: S1 Table — (DOCX) [file pone.0342987.s001.docx]

| **Supplemental Table 1. Summary of Representative Cases** | | | | | | |  |  |
| --- | --- | --- | --- | --- | --- | --- | --- | --- |
|  | OCT Findings | | | | | | | |
|  | Three distinct layers | Fibrous plaques | Fibroatheroma | Fibrocalcific plaque | Microvessels | SSR | Protruding mass regarded  as thrombi | Ruptured plaque |
| Normal Coronary  Arterial Segments  from the Onset | Figure 3 A4, A5, B5 |  |  |  |  |  |  |  |
| Regressed Aneurysms | Figure 4 A4 Figure 5 B5 | Figure 3 A1−3 Figure 5 B6 | Figure 5 A1, A3 | Figure 4 A1 Figure 5 A4, A5 | Figure 4 A2 Figure 5 A6 |  | Figure 5 A5 | Figure 5 A2 |
| Persistent Aneurysms |  |  | Figure 3 B2 Figure 5 B4 | Figure 5 B2 | Figure 5 B3 | Figure 3 B2 Figure 5 B1 Figure 6 A5 |  |  |
| Localized Stenotic Lesions |  |  | Figure 6 A4 | Figure 6 A1-3 | Figure 6 A1, B3 | Figure 6 A5 | Figure 6 A2, B4 | Figure 6 A2 |
