# Supplementary material for: Optical coherence tomography-derived coronary vessel wall abnormalities in adults long after Kawasaki disease
Source: PLoS One. 2026 Feb 25;21(2):e0342987. doi: 10.1371/journal.pone.0342987 (PMC12935235; doi:10.1371/journal.pone.0342987)
Supplement: S1 File — Patient 3, who had thrombi on fibrocalcific plaque with microvessels and no atheroma in the left coronary artery, was accompanied by subendocardial infarction in cardiac MR imaging (S1 Fig). Patient 7, with a small ruptured plaque on fibroatheroma, had neither cardiac events nor MR-derived silent myocardial infarction in the corresponding segment (S2 Fig). In the MDCT study, the presence of MDCT-derived calcified plaque was in parallel with the severity of CAG-derived luminal lesions (S3 Fig). (DOCX) [file pone.0342987.s002.docx]

**Supplement I: Specific Cases of Luminal Thrombi or Ruptured Plaques.**Patient 3, who had thrombi on fibrocalcific plaque with microvessels and no atheroma in the left coronary artery, was accompanied by subendocardial infarction in cardiac MR imaging (Supplemental Figure 1). Patient 7, with a small ruptured plaque on fibroatheroma, had neither cardiac events nor MR-derived silent myocardial infarction in the corresponding segment (Supplemental Figure 2). In the MDCT study, the presence of MDCT-derived calcified plaque was in parallel with the severity of CAG-derived luminal lesions (Supplemental Figure 3).
