# Supplementary figures and images for: Optical coherence tomography-derived coronary vessel wall abnormalities in adults long after Kawasaki disease
Source: PLoS One. 2026 Feb 25;21(2):e0342987. doi: 10.1371/journal.pone.0342987 (PMC12935235; doi:10.1371/journal.pone.0342987)

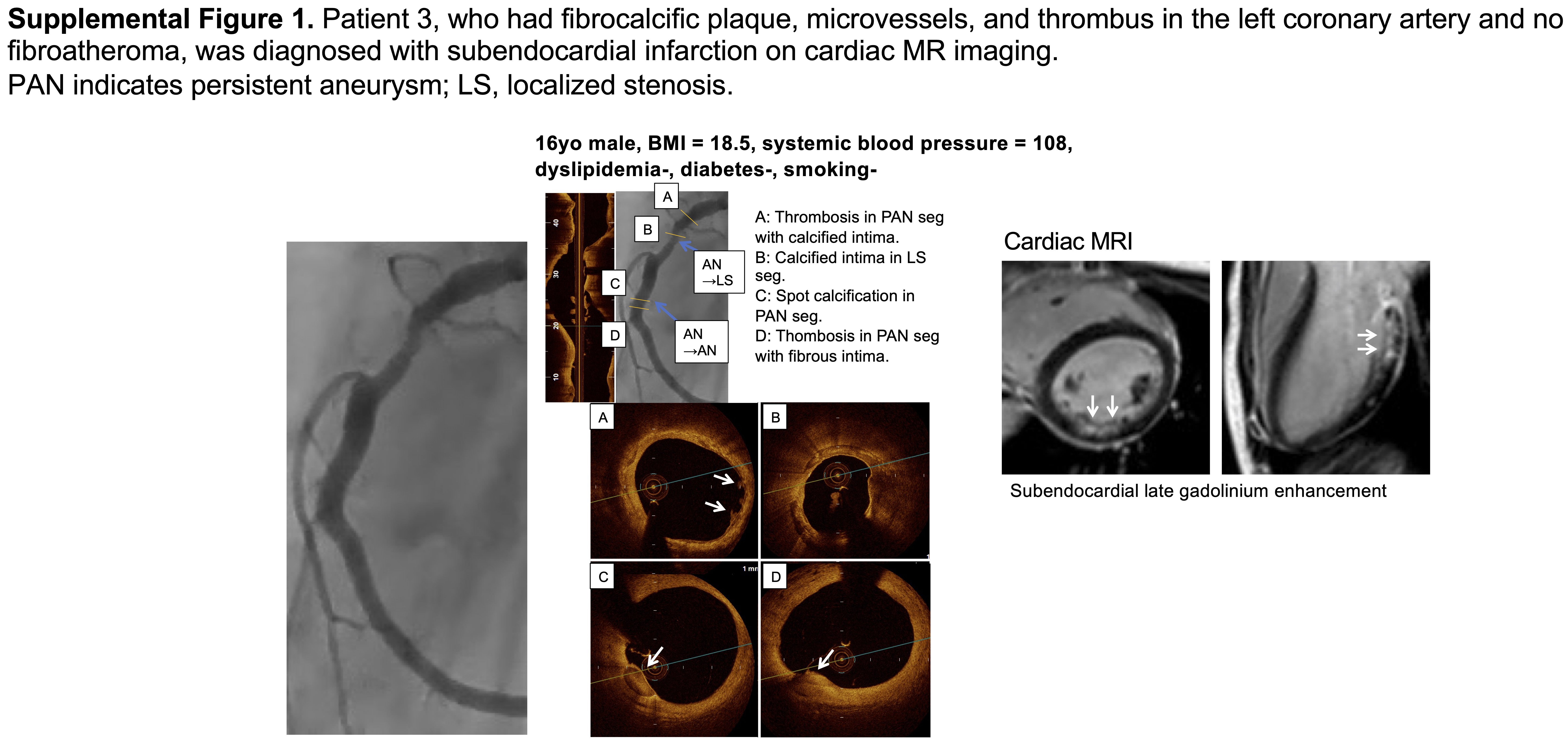

Supplement: S1 Fig — Patient 3, who had fibrocalcific plaque, microvessels, and thrombus in the left coronary artery and no fibroatheroma, was diagnosed with subendocardial infarction on cardiac MR imaging. PAN indicates persistent aneurysm; LS, localized stenosis. (JPG) [file pone.0342987.s003.jpg]

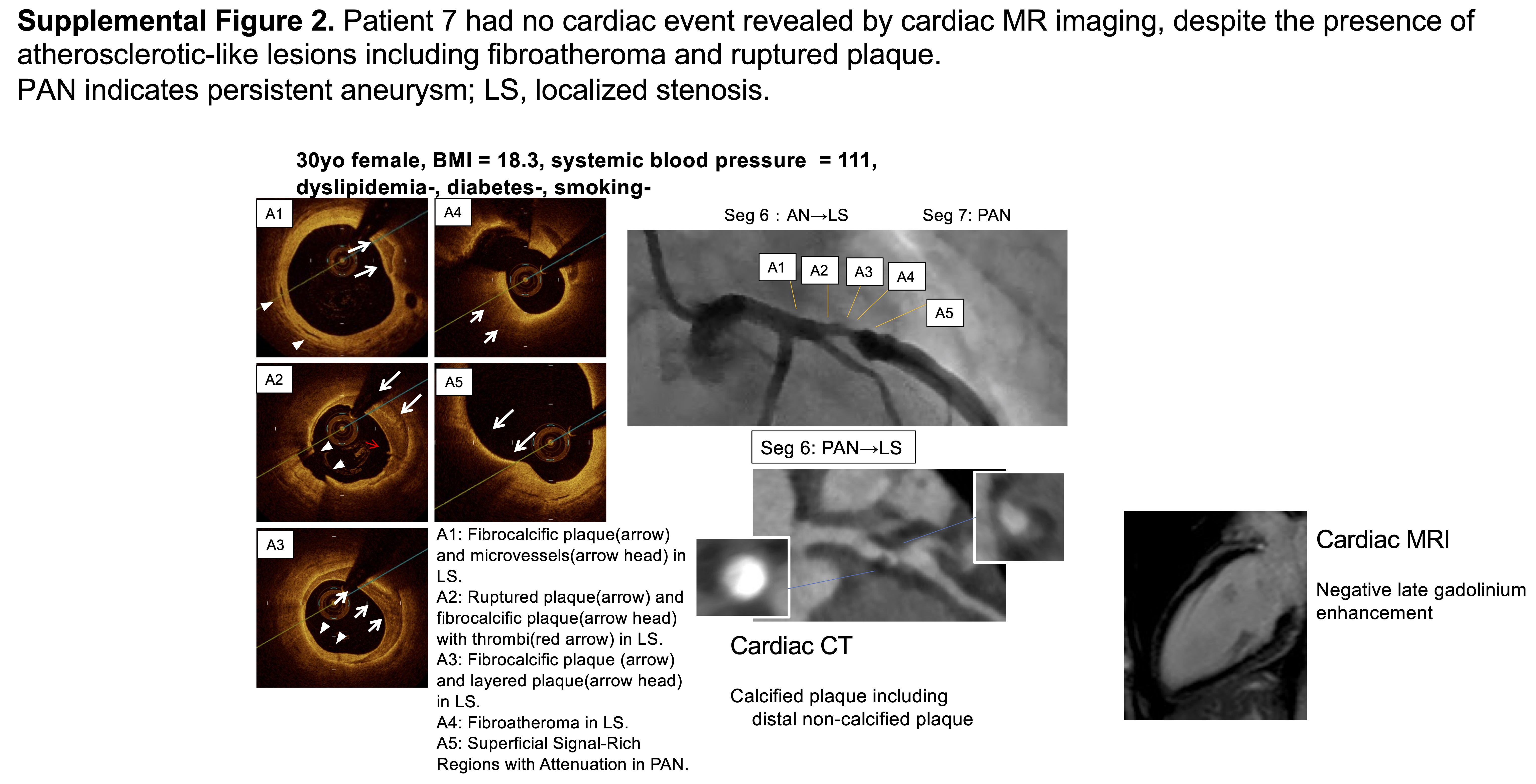

Supplement: S2 Fig — Patient 7 had no cardiac event revealed by cardiac MR imaging, despite the presence of atherosclerotic-like lesions including fibroatheroma and ruptured plaque. PAN indicates persistent aneurysm; LS, localized stenosis. (JPG) [file pone.0342987.s004.jpg]
